# Characteristics and outcomes of patients with COVID-19 at high risk of disease progression receiving sotrovimab, oral antivirals, or no treatment: a retrospective cohort study

**DOI:** 10.1186/s12879-024-09576-7

**Published:** 2024-07-04

**Authors:** Myriam Drysdale, Holly Tibble, Vishal Patel, Daniel C. Gibbons, Emily J. Lloyd, William Kerr, Calum Macdonald, Helen J. Birch, Aziz Sheikh

**Affiliations:** 1 Value Evidence and Outcomes, GSK House, 980 Great West Road, Brentford, Middlesex TW89GS UK; 2 Usher Institute, University of Edinburgh, Scotland, UK; 3 Global Medical Affairs, GSK, Brentford, UK

**Keywords:** COVID-19, Sotrovimab, Molnupiravir, Nirmatrelvir/ritonavir, Omicron BA.1, Omicron BA.2, Omicron BA.5, Oral antivirals, Monoclonal antibody

## Abstract

**Background:**

The clinical benefit of coronavirus disease 2019 (COVID-19) treatments against new circulating variants remains unclear. We sought to describe characteristics and clinical outcomes of highest risk patients with COVID-19 receiving early COVID-19 treatments in Scotland.

**Methods:**

Retrospective cohort study of non-hospitalized patients diagnosed with COVID-19 from December 1, 2021–October 25, 2022, using Scottish administrative health data. We included adult patients who met ≥ 1 of the National Health Service highest risk criteria for early COVID-19 treatment and received outpatient treatment with sotrovimab, nirmatrelvir/ritonavir or molnupiravir, or no early COVID-19 treatment. Index date was defined as the earliest of COVID-19 diagnosis or early COVID-19 treatment. Baseline characteristics and acute clinical outcomes in the 28 days following index were reported. Values of ≤ 5 were suppressed.

**Results:**

In total, 2548 patients were included (492: sotrovimab, 276: nirmatrelvir/ritonavir, 71: molnupiravir, and 1709: eligible highest risk untreated). Patients aged ≥ 75 years accounted for 6.9% (*n* = 34/492), 21.0% (*n* = 58/276), 16.9% (*n* = 12/71) and 13.2% (*n* = 225/1709) of the cohorts, respectively. Advanced renal disease was reported in 6.7% (*n* = 33/492) of sotrovimab-treated and 4.7% (*n* = 81/1709) of untreated patients, and ≤ 5 nirmatrelvir/ritonavir-treated and molnupiravir-treated patients. All-cause hospitalizations were experienced by 5.3% (*n* = 25/476) of sotrovimab-treated patients, 6.9% (*n* = 12/175) of nirmatrelvir/ritonavir-treated patients, ≤ 5 (suppressed number) molnupiravir-treated patients and 13.3% (*n* = 216/1622) of untreated patients. There were no deaths in the treated cohorts; mortality was 4.3% (*n* = 70/1622) among untreated patients.

**Conclusions:**

Sotrovimab was often used by patients who were aged < 75 years. Among patients receiving early COVID-19 treatment, proportions of 28-day all-cause hospitalization and death were low.

## Introduction

The coronavirus disease 2019 (COVID-19) pandemic, as caused by infection with severe acute respiratory syndrome coronavirus 2 (SARS-CoV-2), was declared by the World Health Organization in March 2020 [1]. Older individuals, immunocompromised patients or those with comorbidities such as cancer, diabetes, advanced renal disease or cardiovascular disease are at increased risk of developing severe COVID-19, which may result in hospitalization or death [2, 3].

In the UK, early COVID-19 treatment with either antivirals (e.g., nirmatrelvir/ritonavir, molnupiravir, remdesivir) or monoclonal antibodies (mAbs; e.g., casirivimab/imdevimab, sotrovimab) has been recommended for people with “highest risk” conditions. At the time of the study, examples of these conditions included solid cancer, advanced renal and liver disease, and human immunodeficiency virus (HIV)/acquired immune deficiency syndrome (AIDS) [4]. The emergence of the pandemic threatened to overwhelm healthcare systems in the UK and around the world. Strategies to minimize hospitalizations due to COVID-19 were key to the public health policy response, and hospitalization was an important outcome in the pivotal clinical trials of COVID-19 therapies [5–7].

Sotrovimab is a dual-action engineered human immunoglobulin G1κ mAb derived from the parental mAb S309, a potent neutralizing mAb directed against the spike protein of SARS-CoV-2 [8–11]. In the COVID-19 Monoclonal antibody Efficacy Trial-Intent to Care Early (COMET-ICE) randomized clinical trial (NCT04545060), sotrovimab (500 mg intravenous dose) was shown to significantly reduce the relative risk of all-cause > 24-h hospitalization or death by 79% compared with placebo in high-risk patients with mild-to-moderate COVID-19 [5]. In December 2021, sotrovimab received conditional marketing authorization in the UK for use in symptomatic adults and adolescents (aged ≥ 12 years and weighing ≥ 40 kg) with acute COVID-19 who did not require supplemental oxygen and were deemed at increased risk of progression to severe COVID-19 [12].

Two oral antivirals, molnupiravir and nirmatrelvir/ritonavir, have been shown to reduce the risk of progression to severe COVID-19 compared with placebo among high-risk patients with mild-to-moderate COVID-19 [6, 7, 13]. In the MOVe-OUT trial (NCT04575597), participants receiving molnupiravir had a lower risk of hospitalization or death through day 29 compared with the placebo group (6.8% vs 9.7%; difference, 3.0 percentage points; 95% CI, − 5.9 to − 0.1) [7]. In the EPIC-HR trial (NCT04960202), nirmatrelvir/ritonavir significantly reduced the incidence of hospitalization or death by day 28 compared with placebo (difference, 5.81 percentage points; 95% CI, –7.78 to –3.84; relative risk reduction, 88.9%) [6]. Molnupiravir and nirmatrelvir/ritonavir received conditional marketing authorization in November 2021 and December 2021, respectively, for use in patients with COVID-19 who were deemed at increased risk of disease progression [14, 15].

During the study period (December 2021–October 2022), National Health Service (NHS) guidelines recommended sotrovimab and nirmatrelvir/ritonavir as first-line treatment options, and molnupiravir as a third-line option [16]. It should be noted that nirmatrelvir/ritonavir may be contraindicated for certain highest-risk patients, including those with advanced renal disease or receiving ritonavir-containing medication for HIV/AIDS [17]. These guidelines apply to all parts of the UK, including Scotland [18].

### Aim

Here, we describe real-world use of early COVID-19 treatments (including patient characteristics and clinical outcomes) for the management of non-hospitalized patients with COVID-19 at highest risk of developing severe disease in Scotland.

## Methods

### Study design and data source

This retrospective cohort study followed STROBE and RECORD reporting guidelines. We used data from administrative health datasets managed by Public Health Scotland and National Records of Scotland, linked and pseudonymized by the electronic Data Research and Innovation Service.

The study cohort was drawn from the Scottish general practitioner-registered population living within six health boards (i.e. Ayrshire & Arran, Dumfries & Galloway, Forth Valley, Greater Glasgow & Clyde, Lanarkshire and Lothian) that used the Hospital Electronic Prescribing and Medicines Administration (HEPMA) system for recording administration and prescription of COVID-19 therapies.

The index date (Day 1) was defined as the earliest date of a confirmed COVID-19 diagnosis (via reverse transcriptase polymerase chain reaction [RT-PCR] or lateral flow test [LFT]), or treatment date during the study period. Patients were followed up for 28 days (Day 28) from index (defined as the acute period), during which patient outcomes were evaluated. We included patients diagnosed with COVID-19 from December 1, 2021–October 25, 2022. The baseline period was defined as the 2 years prior to index for secondary care events and 1 year prior to index for general practitioner prescriptions.

### Study population

Non-hospitalized patients (defined as having treatment for COVID-19 initiated in an outpatient or community setting) were eligible for inclusion if they were aged ≥ 18 years on the index date; had a COVID-19 diagnosis/positive SARS-CoV-2 RT-PCR or LFT; lived within 1 of the 6 geographical zones attached to a Scottish health board that used the HEPMA prescribing system; met ≥ 1 of the NHS highest-risk conditions criteria for receiving early treatment with sotrovimab, nirmatrelvir/ritonavir or molnupiravir (as defined by the presence of diagnosis codes) (Table 1); and received outpatient treatment with sotrovimab, nirmatrelvir/ritonavir, or molnupiravir, or received no early COVID-19 treatment.

**Table 1:** Highest and high-risk conditions criteria

Highest risk conditions	High-risk conditions
Down syndrome	Age ≥ 70 years (without highest-risk conditions)
Solid cancer	Long-term respiratory conditions
Hematologic disease and stem-cell transplant recipients	Chronic heart disease^a^
Advanced renal disease	Chronic kidney disease
Liver disease	Chronic liver disease
IMID	Chronic neurologic condition
Immune deficiencies	Diabetes
HIV/AIDS	Weakened immune system caused by medical condition or medication^a^
Solid-organ transplant	Obesity (class III)^a^
Rare neurologic conditions	Pregnancy
	Severe respiratory conditions^a^
	Rare disease and inborn errors of metabolism^a^

^a^ No data available for this condition

*AIDS* acquired immune deficiency syndrome, *HIV* human immunodeficiency virus, *IMID* immune-mediated inflammatory disease

At the time of study, the NHS highest-risk criteria were Down’s syndrome, solid cancer, hematologic diseases (including cancers), advanced renal disease, advanced liver disease, immune-mediated inflammatory disease (IMID), immune deficiencies, HIV/AIDS, solid-organ and stem-cell transplant recipients, and rare neurologic conditions [4]. These highest-risk criteria were evaluated during the baseline period.

Patients were excluded if they received more than one COVID-19 treatment (sotrovimab, nirmatrelvir/ritonavir, molnupiravir, or remdesivir) in an outpatient setting during the acute period. Patients were also excluded if they received remdesivir as an early treatment in an outpatient setting, or initiated any COVID-19 treatment while in an inpatient setting (defined as overnight admission on the day of or prior to treatment, and discharge after the day of treatment). These latter two criteria were intended to ensure that only non-hospitalized patients with mild-to-moderate COVID-19 were included.

### Study outcomes

The primary outcomes of this study were the proportions of patients with all-cause and COVID-19-related hospitalizations during the acute period (28 days following index). COVID-19-related hospitalizations were defined as any non-elective hospital visit for which COVID-19 was listed in the primary diagnosis field (among patients in whom the hospitalization episode was complete, i.e. discharge had occurred and clinical coding was complete).

Secondary outcomes included the number of all-cause and COVID-19-related inpatient hospitalization days, the proportion of patients with a critical care admission as part of hospitalization, the proportion of patients requiring non-invasive ventilation and mechanical ventilation, and the proportion of deaths during the acute period.

For all outcomes, patients were excluded if there was less than 45 days between the index diagnosis and data extraction censoring dates (regardless of whether they died in this interval) for the outcomes data (October 6, 2022). Forty-five days is the period recommended by Public Health Scotland to account for a reporting lag in the datasets used.

Patient characteristics were also recorded, including age, sex, COVID-19 vaccination status, and comorbidity history. Cohorts were described in relation to “highest-risk” conditions that made patients eligible for early treatment with sotrovimab, nirmatrelvir/ritonavir, or molnupiravir, as mentioned above. Additionally, the cohorts were described in relation to other “high-risk” conditions that may predispose patients to severe COVID-19 outcomes (Table 1).

Outcomes were reported for the following cohorts: Cohort 1, patients receiving early treatment with sotrovimab; Cohort 2, patients receiving early treatment with nirmatrelvir/ritonavir; Cohort 3, patients receiving early treatment with molnupiravir; and Cohort 4, patients at highest risk who received no early COVID-19 treatment.

**Fig. 1:**
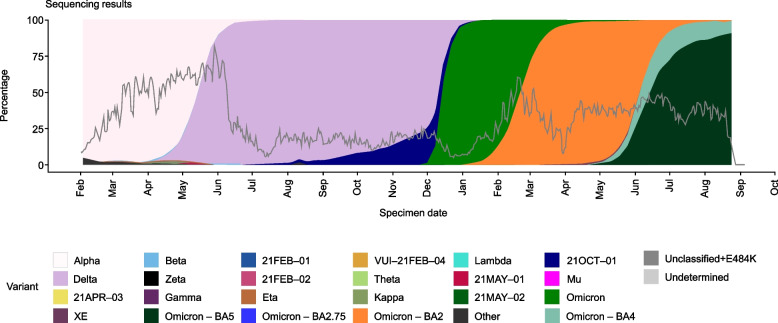
SARS-CoV-2 variant predominance from February 2021 to September 2022. UK Health Security Agency. SARS-CoV-2 variants of concern and variants under investigation in England. Technical briefing 45. 2022. Reprinted from: https://assets.publishing.service.gov.uk/government/uploads/system/uploads/attachment_data/file/1115071/Technical-Briefing-45-9September2022.pdf [19]. Contains public sector information licensed under the Open Government Licence v3.0: https://www.nationalarchives.gov.uk/doc/open-government-licence/version/3/. Accessed 17 Aug 2023. SARS-CoV-2*,* severe acute respiratory syndrome coronavirus 2

A subgroup analysis was also conducted. We described 28-day COVID-19-related hospitalization among sotrovimab-treated patients (Cohort 1) and those without any early COVID-19 treatments (Cohort 4) during the periods of Omicron BA.1 (December 1, 2021–February 28, 2022), Omicron BA.2 (March 1–May 31, 2022) and Omicron BA.5 (June 1–September 30, 2022) subvariant predominance in the UK (Fig. 1) [19]. These analyses were not performed for patients treated with nirmatrelvir/ritonavir or molnupiravir due to small sample sizes. Due to low sequencing rates, periods of most prevalent circulating variants were used as a proxy for the infecting variant.

### Data analysis

Continuous variables (e.g. age) were summarized using mean, standard deviation, median, interquartile range, and range. Categorical variables (e.g. sex) were described using frequencies and percentages. Due to the study’s information governance and data suppression rules, counts of between 0 and 5 were suppressed and are reported as *n* =  ≤ 5 throughout (unless they were structural zeros caused by inclusion/exclusion criteria). This suppression did not apply to mortality data.

## Results

### Patient demographics and baseline characteristics

**Fig. 2:**
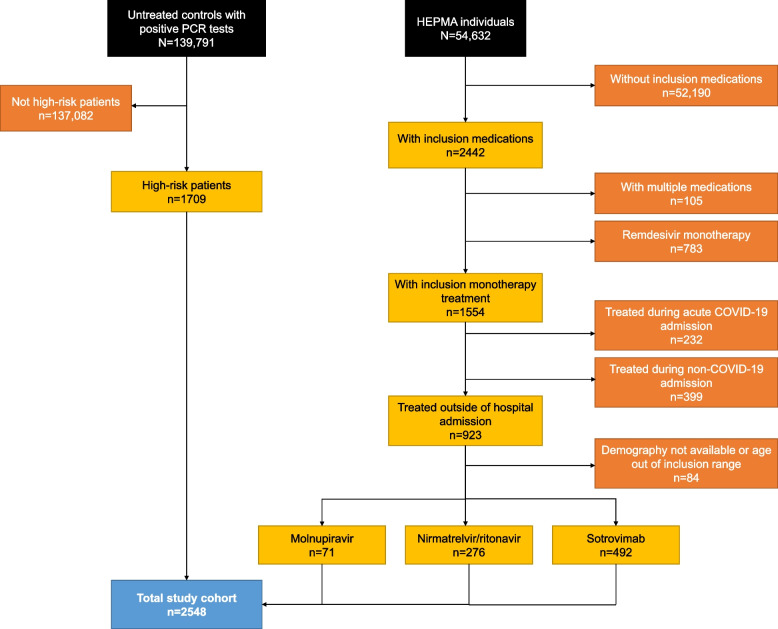
Flow diagram of patient cohort inclusion/exclusion criteria. COVID-19, coronavirus disease 2019; HEPMA, Hospital Electronic Prescribing and Medicines Administration; PCR, polymerase chain reaction

Following application of the eligibility criteria, demographics and baseline characteristics were available for 2548 patients, including 492 patients treated with sotrovimab, 276 patients treated with nirmatrelvir/ritonavir, 71 patients treated with molnupiravir, and 1709 eligible highest-risk untreated patients (Fig. 2). Baseline characteristics are reported in Table 2.

**Table 2:** Patient characteristics

Characteristic	Sotrovimab (*n* = 492)	Nirmatrelvir/ritonavir (*n* = 276)	Molnupiravir (*n* = 71)	Untreated (*n* = 1709)
Age (years)				
Mean (SD)	54.6 (15.0)	60.9 (17.3)	59.6 (14.0)	55.1 (17.9)
Median (IQR)	55 (44–65)	62 (50–73)	60 (49.5–69.5)	57 (41–69)
Age group (years), n (%)				
18–54	242 (49.2)	95 (34.4)	22 (31.0)	783 (45.8)
55–64	121 (24.6)	61 (22.1)	20 (28.2)	369 (21.6)
65–74	95 (19.3)	62 (22.5)	17 (23.9)	332 (19.4)
≥ 75	34 (6.9)	58 (21.0)	12 (16.9)	225 (13.2)
Female sex, n (%)	286 (58.1)	164 (59.4)	42 (59.2)	949 (55.5)
Number of vaccinations, n (%)				
0–2	21 (4.3)	19 (6.9)	≤ 5	315 (18.4)
3	281 (57.1)	126 (45.7)	41 (57.7)	1036 (60.6)
4	160 (32.5)	88 (31.9)	23 (32.4)	283 (16.6)
≥ 5	30 (6.1)	43 (15.6)	≤ 5	75 (4.4)
Time since last vaccination (days), n (%)				
1–60	164 (33.3)	37 (13.4)	17 (23.9)	369 (21.6)
61–120	204 (41.5)	56 (20.3)	28 (39.4)	542 (31.7)
121–180	74 (15.0)	88 (31.9)	10 (14.1)	371 (21.7)
≥ 181	50 (10.2)	95 (34.4)	16 (22.5)	427 (25.0)
Highest risk conditions, n (%)^a^				
Solid cancer	45 (9.1)	16 (5.8)	≤ 5	501 (29.3)
Hematologic disease, sickle cell disease and stem-cell transplant recipients	28 (5.7)	15 (5.4)	≤ 5	89 (5.2)
Advanced renal disease	33 (6.7)	≤ 5	≤ 5	81 (4.7)
Liver disease	≤ 5	≤ 5	≤ 5	23 (1.3)
IMID	31 (6.3)	12 (4.3)	≤ 5	964 (56.4)
Immune deficiencies	≤ 5	≤ 5	≤ 5	7 (0.4)
HIV/AIDS	≤ 5	≤ 5	≤ 5	≤ 5
Solid-organ transplant	≤ 5	≤ 5	≤ 5	≤ 5
Rare neurologic conditions	9 (1.8)	≤ 5	≤ 5	72 (4.2)
Patients with no highest risk diagnoses for developing COVID-19 complications, n (%)	353 (71.7)	235 (85.1)	61 (85.9)	0
High-risk conditions, n (%)^b^				
Age ≥ 70 years (and no highest risk conditions)	56 (11.4)	84 (30.4)	17 (23.9)	0
Chronic respiratory conditions	15 (3.0)	8 (2.9)	≤ 5	49 (2.9)
Other chronic kidney disease	≤ 5	≤ 5	≤ 5	≤ 5
Other chronic liver disease	≤ 5	≤ 5	≤ 5	8 (0.5)

Due to information governance and data suppression rules used in the study, counts of between 0 and 5 were suppressed and are reported as *n* =  ≤ 5 throughout, excluding structural zeros (due to inclusion/exclusion criteria)

^a^No cases of Down syndrome were identified from the available data; ^b^ No cases of pregnancy, chronic, moderate-risk neurologic conditions, or diabetes were identified from the available data

*AIDS* acquired immune deficiency syndrome, *COVID-19* coronavirus disease 2019; HIV, human immunodeficiency virus, *IMID* immune-mediated inflammatory disease, *IQR* interquartile range, *SD* standard deviation

Patients aged ≥ 75 years accounted for 6.9% (*n* = 34/492) of the sotrovimab-treated cohort, 21.0% (*n* = 58/276) of the nirmatrelvir/ritonavir-treated cohort, 16.9% (*n* = 12/71) of the molnupiravir-treated cohort, and 13.2% (*n* = 225/1709) of untreated patients.

A high proportion of patients receiving an early COVID-19 treatment did not have an identifiable highest-risk condition in the database (71.7% for sotrovimab [*n* = 353/492], 85.1% for nirmatrelvir/ritonavir [*n* = 235/276], 85.9% for molnupiravir [*n* = 61/71], and 0% for untreated patients [resulting from the cohort’s inclusion criteria]). A high percentage of untreated patients had solid cancer (29.3%, *n* = 501/1709) and IMID (56.4%, *n* = 964/1709), while comparatively lower percentages were reported for other highest-risk comorbidities among this cohort (Table 2). In the treated cohorts, most patients did not have data available on highest-risk comorbidities, and low proportions of such conditions were therefore reported. Among sotrovimab-treated patients, 9.1% (*n* = 45/492) had solid cancer and 6.3% (*n* = 31/492) had IMID. Solid cancer was the most frequently reported highest-risk condition for nirmatrelvir/ritonavir-treated patients (5.8%, *n* = 16/276), and each of the highest-risk comorbidities were reported in five or fewer molnupiravir-treated patients. Advanced renal disease was reported for 6.7% (*n* = 33/492) of sotrovimab-treated patients and 4.7% (*n* = 81/1709) of untreated patients. Five or fewer patients treated with nirmatrelvir/ritonavir (*n* =  ≤ 5/276) and molnupiravir (*n* =  ≤ 5/71) had advanced renal disease. Due to the high level of missing data, the proportions of patients with highest-risk comorbidities could not be compared between cohorts.

### Acute period outcomes

In total, 8.5% (*n* = 216/2548) of patients did not have sufficient observation time (45 days, including after death, from index to data extraction date) to assess acute period outcomes (sotrovimab: *n* = 16/492 [3.3%]; nirmatrelvir/ritonavir: *n* = 101/276 [36.6%]; molnupiravir: *n* = 12/71 [16.9%]; untreated: *n* = 87/1709 [5.1%]). Fewer than five patients with insufficient observation time died.

The percentage of patients who experienced an all-cause hospitalization was 5.3% (*n* = 25/476) for sotrovimab-treated patients and 6.9% (*n* = 12/175) for nirmatrelvir/ritonavir-treated patients (Table 3). Five or fewer molnupiravir-treated patients (*n* =  ≤ 5/59) experienced an all-cause hospitalization. For untreated patients, the percentage of all-cause hospitalizations was 13.3% (*n* = 216/1622). The median (interquartile range) number of days in hospital due to any cause during the acute period was 5.0 (2–9) for sotrovimab-treated patients, 3.5 (2–9.5) for patients treated with nirmatrelvir/ritonavir, and 6.0 (3–14) for untreated patients. Median length of stay could not be reported for molnupiravir due to low patient numbers. Five or fewer patients in all cohorts required non-invasive ventilation or mechanical ventilation. Critical care admissions were experienced by five or fewer patients in each of the treated cohorts and 10 (0.6%) untreated patients.

**Table 3:** Acute period all-cause and COVID-19-related outcomes

Outcome	Sotrovimab (*n* = 476)	Nirmatrelvir/ritonavir (*n* = 175)	Molnupiravir (*n* = 59)	Untreated (*n* = 1622)
**All-cause**				
Inpatient (overnight) hospitalization, n (%)	25 (5.3)	12 (6.9)	≤ 5	216 (13.3)
Inpatient hospitalization days				
Mean (SD)	7.0 (6.0)	8.8 (11.1)	NR	10.8 (14.0)
Median (IQR)	5 (2–9)	3.5 (2–9.5)		6 (3–14)
Critical care admission, n (%)	≤ 5	≤ 5	≤ 5	10 (0.6)
Non-invasive ventilation, n (%)	≤ 5	≤ 5	≤ 5	≤ 5
Mechanical ventilation, n (%)	≤ 5	≤ 5	≤ 5	≤ 5
Inpatient (overnight) hospitalization with COVID-19 recorded in any diagnosis field, n (%)	≤ 5	≤ 5	≤ 5	66 (4.1)
Death, n (%)	0	0	0	70 (4.3)
**COVID-19-related**				
Inpatient (overnight) hospitalization (primary diagnosis), n (%)	≤ 5	≤ 5	≤ 5	48 (3.0)
Inpatient hospitalization days				
Mean (SD)	NR	NR	NR	8.4 (7.4)
Median (IQR)				6 (3–12)
Critical care admission, n (%)	≤ 5	≤ 5	≤ 5	≤ 5
Non-invasive ventilation, n (%)	≤ 5	≤ 5	≤ 5	≤ 5
Mechanical ventilation, n (%)	≤ 5	≤ 5	≤ 5	≤ 5
Death, n (%)	0	0	0	13 (0.8)

The hospitalizations dataset ran from December 1, 2019 to October 11, 2022, the intensive care unit dataset ran from December 1, 2021 to October 23, 2022, and the deaths dataset ran from December 2, 2021 to October 6, 2022. These three outcomes datasets were censored to align with the earliest dataset (to October 6, 2022) for consistency

Due to information governance and data suppression rules used in the study, counts of between 0 and 5 were suppressed and are reported as *n* =  ≤ 5 throughout, except for mortality data, which does not require suppression

*COVID-19* coronavirus disease 2019, *IQR* interquartile range, *NR* not reported, *SD* standard deviation

Five or fewer patients treated with each of sotrovimab, nirmatrelvir/ritonavir, and molnupiravir experienced COVID-19-related hospitalizations during the acute period (Table 3). For untreated patients, the percentage of COVID-19-related hospitalizations was 3.0% (*n* = 48/1622). Five or fewer patients in all cohorts required non-invasive ventilation or mechanical ventilation, or experienced critical care admission.

There were no deaths within 28 days of index for patients treated with sotrovimab, nirmatrelvir/ritonavir or molnupiravir. Mortality was 4.3% (*n* = 70/1622) in untreated patients, of whom 13 patients (18.6%) had COVID-19 as the primary cause (Table 3).

### COVID-19-related hospitalizations during BA.1, BA.2, and BA.5 predominance

During BA.1 predominance, 283 patients were treated with sotrovimab and 721 received no treatment (Table 4). Similar to the overall analysis, five or fewer sotrovimab-treated patients (*n* =  ≤ 5/283) experienced a COVID-19-related hospitalization. The percentage for untreated patients was 3.1% (*n* = 22/721).

**Table 4:** Acute period COVID-19-related hospitalizations during periods of Omicron BA.1, BA.2, and BA.5 predominance

	**Sotrovimab**	**Untreated**
**BA.1** (December 1, 2021 to February 28, 2022)	≤ 5/283	22/721 (3.1%)
**BA.2** (March 1, 2022 to May 31, 2022)	≤ 5/144	14/625 (2.2%)
**BA.5** (June 1, 2022 to September 30, 2022)	≤ 5/49	12/276 (4.3%)

Due to information governance and data suppression rules used in the study, counts of between 0 and 5 were suppressed and are reported as *n* =  ≤ 5 throughout

*COVID-19* coronavirus disease 2019

During BA.2 predominance, 144 patients were treated with sotrovimab and 625 received no treatment (Table 4). Five or fewer sotrovimab-treated patients (*n* =  ≤ 5/144) and 2.2% (n = 14/625) of untreated patients had COVID-19-related hospitalizations.

During BA.5 predominance, 49 patients were treated with sotrovimab and 276 received no treatment (Table 4). Five or fewer sotrovimab-treated patients (n =  ≤ 5/49) and 4.3% (*n* = 12/276) of untreated patients had COVID-19-related hospitalizations.

## Discussion

This study described the characteristics and severe clinical outcomes of non-hospitalized patients who received early COVID-19 treatment in Scotland from December 1, 2021–October 25, 2022, or those who were likely eligible but did not receive treatment. Our findings indicate that sotrovimab was used among patients who were slightly younger (6.9% were aged ≥ 75 years vs 21.0% for nirmatrelvir/ritonavir and 16.9% for molnupiravir). We also found that patients who received early COVID-19 treatment with sotrovimab or antivirals experienced low levels of 28-day hospitalization. Low levels of all-cause death were also observed.

The observed characteristics of sotrovimab-treated patients differed to those described in a similar real-world study conducted in another part of the UK [20]. In a retrospective cohort study of non-hospitalized patients who received early treatment for, or were diagnosed with, COVID-19 in Northwest London from December 1, 2021–May 31, 2022, Patel et al. reported descriptive results for patients treated with sotrovimab, nirmatrelvir/ritonavir, or molnupiravir. Sotrovimab was found to be used most often among older patients with multiple comorbidities that increased their risk of severe COVID-19, such as advanced renal disease [20]. In the present study, we found that sotrovimab-treated patients were younger than the other treated cohort. Of note, we observed a particularly high number of untreated patients with IMID (56.4%). This suggests that the effective eligibility criteria may have been narrower than the definitions used herein, with certain IMIDs associated with higher risk and real-world treatment allocation reflecting this. Alternatively, it may indicate genuine clinical differences between the treated and untreated cohorts.

We found that the proportion of all-cause hospitalizations was low for patients treated with sotrovimab and antivirals. These results are supported by other real-world studies. Patel et al. reported low all-cause hospitalizations among patients treated with sotrovimab, nirmatrelvir/ritonavir, and molnupiravir, with low hospitalization rates for sotrovimab being consistent among patients with advanced renal disease, those aged 18–64 years and ≥ 65 years, and across periods of Omicron BA.1, BA.2, and BA.5 predominance (through July 2022) [20]. Our results for the overall cohort are also similar to those of a recent retrospective cohort study of patients presumed to be treated with sotrovimab in England, where 4.6% of patients experienced an all-cause hospitalization [21].

A recent publication investigated outcomes among patients on kidney replacement therapy in Scotland and England using OpenSAFELY and UK Renal Registry data. Among this population, 1.1% (*n* = 21/1852) of sotrovimab-treated patients experienced 28-day COVID-19-related hospitalization or death compared with 3.3% (*n* = 17/515) of molnupiravir-treated patients [22]. In an analysis of sotrovimab vs molnupiravir in England (conducted from December 2021 to February 2022), 0.1% (*n* = 32/3331) of sotrovimab-treated patients and 2.0% (*n* = 55/2689) of molnupiravir-treated patients experienced 28-day COVID-19-related hospitalization or death [23]. In a US study conducted during Delta and early Omicron variant predominance, 2.67% (*n* = 418/15,633) of sotrovimab-treated patients experienced 30-day all-cause hospitalization and 0.08% (*n* = 13/15,633) died, compared with 5.57% (*n* = 84,307/1,514,868) and 0.54% (*n* = 8167/1,514,868), respectively, of patients who did not receive mAb treatment [24]. In a further study conducted from February to October 2022, very similar proportions of patients treated with sotrovimab and nirmatrelvir/ritonavir experienced 28-day COVID-19-related hospitalization or death [25]. In another recent study, the OpenSAFELY platform was used to emulate target trials to estimate the effectiveness of sotrovimab vs no treatment during BA.1 and BA.2 predominance. Estimated hazard ratios for 28-day COVID-19-related hospitalization or death were 0.76 (95% confidence interval [CI]: 0.66–0.89) during BA.1 and 0.92 (95% CI: 0.79–1.06) during BA.2 for sotrovimab vs no treatment [26].

We previously assessed the uptake of mAbs and antiviral therapies in Scotland, and discussed whether these treatments were used as recommended [27]. We reported that only around half of eligible patients received a mAb/antiviral for COVID-19, but the vast majority of patients who received treatment did so within the recommended timeframe. In this study, we also found that some eligible patients (based on COVID-19 diagnosis and high-risk comorbidities) were untreated; the reasons for this were not ascertained as part of this study but are of interest for future research.

We also found that five or fewer sotrovimab-treated patients experienced COVID-19-related hospitalizations across periods of BA.1, BA.2 and BA.5 predominance. Due to data suppression rules and small patient numbers (particularly with decreasing sotrovimab use during BA.5), interpretation of these results is challenging, particularly during BA.2 and BA.5 predominance. The risk of ecologic bias should be noted, as we did not have access to sequencing data to confirm each patient’s variant.

It should be noted that these descriptive results require confirmation with formal statistical testing adjusting for confounding factors. In the current study, formal comparison was not considered appropriate for several reasons. Due to the relatively small sample size for some groups, and low numbers of events observed (resulting in suppressed numbers), it was highly unlikely that there would be sufficient power to show statistical significance. More importantly, despite it being an inclusion criterion, for a high number of patients in the treatment groups (> 70%) their highest-risk conditions could not be identified in the database; this meant it was impossible to adequately adjust for potential confounders.

This study has several limitations. Firstly, we present descriptive analyses with no adjustment for differences in patient characteristics between cohorts, meaning that results may be subject to bias and confounding. Further, the limited sample size (particularly for molnupiravir) led to frequent suppression of small values. We did not collect data on COVID-19 severity and symptoms at disease onset, and therefore cannot confirm the cohorts were comparable in this regard. It is possible that the sotrovimab-treated cohort had mild-to-moderate COVID-19, while some untreated patients may have been asymptomatic, mildly symptomatic, or improving. In addition, 85% of the treated cohort had no identified RT-PCR or (registered) LFT in the 40 days prior to treatment, whereas the untreated cohort were required to have a positive test recorded. These “untested” treated patients were likely to have tested at home and not reported their results. There were limited sequencing data available for included patients, and dominance period for Omicron subvariant was used as a surrogate; hence, there is potential for misclassification of the infecting variant. Also, comorbidities were identified using inpatient admissions and procedures data; conditions treated in specialist departments (including maternity and renal wards) or in primary care could not, therefore, be determined, which may partially explain the level of missing data observed. Finally, there was an imbalance across study cohorts in the proportion of patients from each group who could not be included for analysis due to insufficient observation time (< 45 days); in particular, more than one-third of those in the nirmatrelvir/ritonavir group had an insufficient observation period, which may have impacted our findings. Patients with observation time < 45 days were excluded from the main analysis even if they died; this accounted for less than five patients, and their inclusion in a sensitivity analysis had no impact on study results (data not shown).

## Conclusion

Our findings indicate that among patients who received early COVID-19 treatment with sotrovimab or antivirals in Scotland, low proportions experienced all-cause hospitalizations and death within 28 days of treatment. Sotrovimab was observed to be frequently utilized in patients aged below 75 years old in Scotland. Most treated patients had missing data for their high/highest-risk status and conditions, which reduced the feasibility of conducting a comparative effectiveness analysis to assess the impact of sotrovimab in preventing severe COVID-19 among this population.

## Data Availability

Data for this study (study ID: 2223–0033) are held by the National Services Scotland electronic Data Research and Innovation Service in the National Safe Haven. Restrictions apply to the availability of these data, which were used under license for the current study, and so are not publicly available. Data would be made available from a reasonable request to myriam.g.drysdale@gsk.com.

## Abbreviations

AIDS: Acquired immune deficiency syndrome
CI: Confidence interval
COMET-ICE: COVID-19 Monoclonal antibody Efficacy Trial-Intent to Care Early
COVID-19: Coronavirus disease 2019
HEPMA: Hospital Electronic Prescribing and Medicines Administration
HIV: Human immunodeficiency virus
IMID: Immune-mediated inflammatory disease
IQR: Interquartile range
LFT: Lateral flow test
mAb: Monoclonal antibody
NHS: National Health Service
NR: Not reported
PCR: Polymerase chain reaction
RT-PCR: Reverse transcriptase polymerase chain reaction
SARS-CoV-2: Severe acute respiratory syndrome coronavirus 2
SD: Standard deviation
